# Sexual- und Verhütungsverhalten Jugendlicher und junger Erwachsener in Deutschland: Aktuelle Ergebnisse der repräsentativen Wiederholungsbefragung „Jugendsexualität“

**DOI:** 10.1007/s00103-026-04203-z

**Published:** 2026-02-23

**Authors:** Sara Scharmanski, Luise Dinger

**Affiliations:** Sexualaufklärung, Verhütung und Familienplanung, Bundesinstitut für Öffentliche Gesundheit (BIÖG), Maarweg 149–161, 50825 Köln, Deutschland

**Keywords:** Kondom, Pille, Hormonelle Verhütung, Notfallkontrazeption, Sexualaufklärung, Condom, Pill, Hormonal contraception, Emergency contraception, Comprehensive sexuality education (CSE)

## Abstract

**Hintergrund:**

Seit 1980 führt das Bundesinstitut für Öffentliche Gesundheit (BIÖG; ehemals Bundeszentrale für gesundheitliche Aufklärung, BZgA) regelmäßig die repräsentative Erhebung „Jugendsexualität“ durch. Sie liefert empirische Erkenntnisse zur sexuellen und reproduktiven Gesundheit junger Menschen in Deutschland und dient als Grundlage für zielgruppenspezifische Maßnahmen der Sexualaufklärung und Familienplanung.

**Ziel der Arbeit:**

Auf Basis erster deskriptiver Befunde der 10. Befragungswelle 2025 erfolgt eine zusammenfassende Darstellung des Sexual- und Verhütungsverhaltens von Jugendlichen und jungen Erwachsenen.

**Methoden:**

An der Befragung nahmen insgesamt *N* = 5855 Jugendliche und junge Erwachsene teil. Die Datenerhebung erfolgte im ersten Halbjahr 2025 im Auftrag des BIÖG im Rahmen kombinierter mündlich-schriftlicher Interviews mittels Computer-assisted Personal Interviewing (CAPI).

**Ergebnisse:**

Der Zeitpunkt des ersten heterosexuellen Sex hat sich im Vergleich zu früheren Erhebungen weiter nach hinten verschoben. Der erste Sex findet überwiegend im Rahmen fester Beziehungen und mit Verhütung statt. Wie bereits in der Vorwelle 2019 zeigt sich eine zunehmende kritische Haltung gegenüber hormonellen Verhütungsmethoden, der Rückgang der Pillennutzung scheint sich jedoch bei Jugendlichen nicht nennenswert fortzusetzen. Die Pille und das Kondom sind die am meisten verwendeten Verhütungsmethoden.

**Diskussion:**

Der spätere Einstieg in heterosexuelle Aktivitäten entspricht nationalen und internationalen Befunden. Die 10. Befragungswelle belegt ein überwiegend sicheres und verantwortungsbewusstes Verhütungsverhalten junger Menschen in Deutschland. Dennoch bleibt die Weiterentwicklung zielgruppenspezifischer Präventionsangebote ein wichtiges Handlungsfeld der sexuellen Gesundheitsförderung.

**Zusatzmaterial online:**

Zusätzliche Informationen sind in der Online-Version dieses Artikels (10.1007/s00103-026-04203-z) enthalten.

## Abstract (en)

**Background:**

Since 1980, the Federal Institute of Public Health (BIÖG; formerly Federal Centre for Health Education, BZgA) has regularly conducted the representative survey “Youth Sexuality.” It provides empirical data on the sexual and reproductive health of young people in Germany and serves as a basis for target-group-specific sex education and family planning interventions.

**Aim:**

Based on initial descriptive findings from the 10th trend wave in 2025, a summary of adolescents’ and young adults’ sexual and contraceptive behaviors is presented.

**Methods:**

A total of *N* = 5855 adolescents and young adults participated in the survey. Data collection took place in the first half of 2025 on behalf of BIÖG using a combination of oral and written interviews conducted via computer-assisted personal interviewing (CAPI).

**Results:**

Compared to previous survey waves, the age at first heterosexual intercourse among young people has increased. First sexual experiences predominantly occur within steady relationships and with contraceptive use. As in earlier surveys, a growing critical attitude toward hormonal contraceptive methods is evident; however, the decline in pill use does not appear to be continuing among adolescents. The pill and the condom remain the most commonly used contraceptive methods.

**Discussion:**

The later onset of heterosexual activity aligns with national and international findings. Current trends indicate that young people in Germany predominantly practice safe and responsible contraception. Nonetheless, the continued development of target-group-specific prevention services remains a key priority in sexual health promotion.

## Hintergrund

Das Bundesinstitut für öffentliche Gesundheit (BIÖG) – vormals Bundeszentrale für gesundheitliche Aufklärung (BZgA) – ist im Rahmen des Schwangerschaftskonfliktgesetzes (§ 1 SchKG) gesetzlich beauftragt, bundesweite Konzepte und Maßnahmen zur Sexualaufklärung, Verhütung und Familienplanung zu entwickeln und der Bevölkerung kostenfrei bereitzustellen [1].

Zur Umsetzung dieses gesetzlichen Auftrags entwickelt das BIÖG evidenzbasierte, massen- und personalkommunikative Maßnahmen, die auf die Förderung der sexuellen und reproduktiven Gesundheit unterschiedlicher Zielgruppen ausgerichtet sind [2, 3]. Die Jugendsexualitätsstudie liefert seit 1980 ein zuverlässiges empirisches Fundament, um Entwicklungen in der sexuellen und reproduktiven Gesundheit junger Menschen valide zu erfassen [4–6]. Durch die repräsentative Wiederholungsbefragung wird fortlaufend dokumentiert, wie Jugendliche und junge Erwachsene in Deutschland Sexualität erleben, welche Einstellungen sie vertreten, welche Informationsquellen sie nutzen und wie sich ihr Wissen und Verhalten im Laufe der Zeit verändern.

Im vorliegenden Beitrag werden zentrale Ergebnisse der 10. Befragungswelle 2025 vorgestellt und mit den Ergebnissen der Vorwelle 2019 verglichen. Der Fokus liegt auf aktuellen Entwicklungen beim Einstieg in sexuelle Aktivitäten sowie auf Veränderungen im Verhütungsverhalten Jugendlicher und junger Erwachsener in Deutschland.

## Methoden

Die Methodik der vorliegenden repräsentativen Wiederholungsbefragung blieb über die verschiedenen Erhebungswellen hinweg im Wesentlichen unverändert. Jedoch wurde die Stichprobenanlage mehrfach modifiziert, um gesellschaftliche Veränderungen adäquat abzubilden, wie beispielsweise die zunehmende Migration. So lag der Fokus der Befragung ursprünglich auf Jugendlichen im Alter von 14 bis 17 Jahren ohne Migrationshintergrund. In späteren Befragungswellen wurde die Stichprobe um Personen mit Migrationshintergrund erweitert. Seit 2014 umfasst die Zielgruppe zudem auch junge Erwachsene im Alter von 18 bis 25 Jahren.

### Vorgehen

Im Vorfeld der Befragung wurden die Jugendlichen, ihre Erziehungsberechtigten sowie die jungen Erwachsenen mündlich und schriftlich über das Ziel, den Ablauf der Studie sowie datenschutzrelevante Aspekte informiert. Die Teilnahme war freiwillig und setzte die schriftliche Einwilligung der jungen Erwachsenen bzw. Jugendlichen sowie bei Minderjährigen die Zustimmung einer erziehungsberechtigten Person voraus.

Die Datenerhebung erfolgte von Februar bis Juli 2025 durch das Forschungsinstitut Verian und wurde mittels der CAPI-Methode (Computer-assisted Personal Interviewing) mit mündlichen und schriftlichen Interviewteilen von geschulten Interviewenden durchgeführt. Der Mantelfragebogen wurde in einem Face-to-face-Interview erfasst, während intimere Inhalte, beispielsweise zu ersten sexuellen Erfahrungen, von den Teilnehmenden eigenständig am Tablet bzw. Laptop beantwortet wurden (Selbstausfüllerteil). Die Befragung der Jugendlichen und jungen Erwachsenen wurde der Ethikkommission der Deutschen Gesellschaft für Psychologie (DGPs) vorgelegt und von dieser genehmigt.

Für eine Teilgruppe der Stichprobe (Jugendliche im Alter von 14 bis 17 Jahren ohne Migrationshintergrund) wurde zudem jeweils ein Elternteil mit einem separaten Fragebogen befragt. Für ihre Teilnahme erhielten alle Befragten ein monetäres Incentive zwischen 10 € und 20 € (in Abhängigkeit von Feldsituation und Interviewtyp).

### Stichprobe

Da der gesetzliche Auftrag des BIÖG (§ 1 SchKG) die Prävention ungewollter Schwangerschaften umfasst und damit Verhütung und Familienplanung zentrale Themen dieser Studie sind, erfolgt die Datenerhebung traditionell anhand einer disproportionalen Geschlechterstichprobe. Die erhöhte Anzahl befragter Mädchen und junger Frauen ermöglicht es, spezifische Analysen für diese Teilgruppe auf Basis ausreichend großer Fallzahlen durchzuführen. Die Disproportionalität hinsichtlich der Geschlechterverteilung wird durch eine anschließende Gewichtung ausgeglichen, sodass die Ergebnisse repräsentativ für die Grundgesamtheit sind (siehe Abschn. Statistische Analysen).

In der vorliegenden 10. Befragungswelle war eine geschlechtsneutrale Selbstzuordnung möglich, die insgesamt 43 Personen genutzt haben. Zur Erfassung des bei der Geburt zugewiesenen Geschlechts sowie des Geschlechts, mit dem sich die Teilnehmenden identifizieren, wurde sich an der DIVERGesTOOL Toolbox der Universität Bremen^1^ orientiert. Zusätzlich konnten die Befragten die Anredeform während des Interviews festlegen. Vor dem Hintergrund des Erkenntnisinteresses sowie des gesetzlichen Kontexts der Studie wurden die *n* = 43 Personen ohne Geschlechtszuordnung der Gruppe der männlichen Befragung zugeordnet, da Referenzstatistiken zur Gewichtung zum Zeitpunkt der Durchführung für die vorliegende Analyse nicht vorlagen. In allen geschlechtsspezifischen Auswertungen werden Personen, die eine geschlechtsneutrale Kategorie gewählt haben, aus den Analysen ausgeschlossen.

Die disproportionalen Teilstichproben ergeben sich analog zu den vorangegangenen Wellen 8 und 9 aus der Kombination der 3 Kriterien Geschlecht (weiblich vs. männlich/ohne Geschlechtszuordnung), Altersgruppe (14 bis 17 vs. 18 bis 25 Jahre) und kulturelle Herkunft (mit vs. ohne Migrationshintergrund).

Die Operationalisierung des Bildungsniveaus erfolgte über die besuchte Schule und/oder den angestrebten oder erworbenen Schulabschluss. Ein Migrationshintergrund wurde erfasst, sofern entweder die befragte Person selbst oder mindestens ein Elternteil nicht mit deutscher Staatsbürgerschaft geboren wurde [7].

Die Erhebung erfolgte mit dem Quota-Verfahren [8] unter Berücksichtigung der Merkmale Geschlecht, Alter, Wohnregion, kulturelle Herkunft sowie Bildungsstand (besuchte Schulform bzw. Schulabschluss). Die Quoten basieren auf einer Sonderauswertung des Mikrozensus 2023, die das Statistische Bundesamt (Destatis) auf Anfrage erstellte. Aufgrund des eingesetzten Auswahlverfahrens können keine Aussagen zur Stichprobenausschöpfung getroffen werden. Trotz dieser methodischen Limitationen bietet das gewählte Quota-Verfahren mit Blick auf die Erreichbarkeit der jungen Zielgruppe sowie die Anschlussfähigkeit zu früheren Befragungswellen große forschungspraktische Vorteile.

Insgesamt wurden 5939 vollständige Interviews durchgeführt (Interviewdauer: Median (M) = 35 min, Spannweite: 10–320 min). Daneben haben *N* = 290 Personen den Fragebogen abgebrochen, unvollständig bearbeitet oder die Bearbeitung pausiert und nicht wieder aufgenommen. Diese Datensätze wurden von der Analyse ausgeschlossen. Um eine hohe Datenqualität zu gewährleisten, wurden die Interviews mithilfe des Künstliche-Intelligenz-Tools Inspirient auf Non-Response-Anteile sowie auf Interviewdauer (jeweils in Relation zur Fragebogenlänge) geprüft. Aufgrund auffällig hoher Non-Response-Anteile und/oder kurzer Interviewdauer wurden basierend auf festgelegten Cut-off-Werten 84 Interviews (1,4 %) ausgeschlossen. Die finale Stichprobe umfasst *N* = 5855 Personen, darunter *n* = 2013 Mädchen und *n* = 1489 Jungen im Alter von 14 bis 17 Jahren sowie *n* = 1543 junge Frauen und *n* = 767 junge Männer im Alter von 18 bis 25 Jahren. Weitere ungewichtete Merkmale der Teilstichproben können Tab. 1 entnommen werden.

**Tab. 1:** Merkmale der Teilstichproben der 10. Befragungswelle der Jugendsexualitätsstudie 2025 ohne Designgewichtung (*N* = 5855)

Teilstichprobe		Alter 14–17 Jahre		Alter 18–25 Jahre		Gesamt	
		*n*	%	*n*	%	*n*	%
Geschlecht	Weiblich	2013	57	1543	66	3556	61
	Männlich	1489	42	767	33	2256	39
	Ohne Zuordnung	12	0	31	1	43	1
Migrationshintergrund	Ja	1013	29	771	33	1784	30
	Nein	2496	71	1568	67	4064	69
	Nicht zuzuordnen	5	0	2	0	7	0
Bildungsniveau	Niedrig	374	11	222	10	596	10
	Mittel	1648	47	653	28	2301	39
	Hoch	1463	42	1454	62	2917	50
	Keine Angabe	29	1	12	1	41	1

### Erhebungsinstrument

Der Fragebogen erfasst die Einstellungen und das Verhalten von Jugendlichen und jungen Erwachsenen zu den Themen Aufklärung, Sexualität, Kontrazeption und Familienplanung. Um eine Anschlussfähigkeit an die Vorwellen zu gewährleisten, orientiert sich der Fragebogen weitgehend an den vorangehenden Trendmessungen. Darüber hinaus wurde er basierend auf Erkenntnissen aus den Vorwellen sowie unter Berücksichtigung gesellschaftlicher Entwicklungen aktualisiert und erweitert.^2^

Vor der Erhebung erfolgte ein Pretest des Fragebogens mit *n* = 53 Jugendlichen und jungen Erwachsenen (quotiert nach Geschlecht, Alter, Bildung und Migrationshintergrund). Das daraus gewonnene Feedback der Teilnehmenden und Interviewenden wurde bei der Finalisierung des Fragebogens berücksichtigt.

### Statistische Analysen

Um die Repräsentativität der Ergebnisse für die Grundgesamtheit der Jugendlichen und jungen Erwachsenen in Deutschland sicherzustellen, wurde die anfänglich disproportionale Stichprobe durch Gewichtung in eine proportionale überführt. Zur Berechnung der Gewichte wurden Veröffentlichungen des Statistischen Bundesamtes herangezogen. Dabei wurden kombinierte Regional‑, Geschlechter- und Bildungsgewichte ermittelt. Für Befragte mit Migrationshintergrund wurden zusätzlich Gewichte nach Nationalgruppe berechnet. Die Range der Designgewichte lag zwischen 0,22 und 4,66. Alle im Folgenden berichteten Ergebnisse basieren auf der beschriebenen Designgewichtung.

Die Ergebnisse bieten einen ersten Überblick über die Erkenntnisse der 10. Welle der Jugendsexualitätsstudie 2025. Sofern Langzeittrends abgebildet werden, liegt der Fokus ausschließlich auf der Teilgruppe der 14- bis 17-jährigen Jugendlichen ohne Migrationshintergrund, für die seit 1980 Daten vorliegen. Zur Beschreibung des aktuellen Sexual- und Verhütungsverhaltens wurden deskriptive Analysen herangezogen. Alle statistischen Analysen erfolgten mit der Software IBM SPSS Version 25 (IBM, Armonk, NY, USA).

## Ergebnisse

### Sexuelle Orientierung

76 % der befragten Mädchen und jungen Frauen geben an, ausschließlich heterosexuell orientiert zu sein.^3^ Weitere 12 % nennen eine vorwiegende, jedoch nicht ausschließliche heterosexuelle Orientierung. Unter den männlichen Befragten bezeichnen sich 86 % als ausschließlich heterosexuell und 6 % als vorwiegend, aber nicht ausschließlich heterosexuell. Ein vergleichbarer Anteil weiblicher und männlicher Befragter gibt eine (ausschließlich oder vorwiegende) homosexuelle Orientierung an (weiblich: 3 %, männlich: 3 %). Eine bisexuelle Orientierung wird hingegen etwas häufiger von weiblichen als von männlichen Befragten angegeben (weiblich: 8 %, männlich: 3 %), wobei dies bei beiden Geschlechtern überwiegend im Erwachsenenalter genannt wird.

### Sexualverhalten

#### Erfahrungen mit hetero- bzw. homosexuellem Sex

**Abb. 1:**
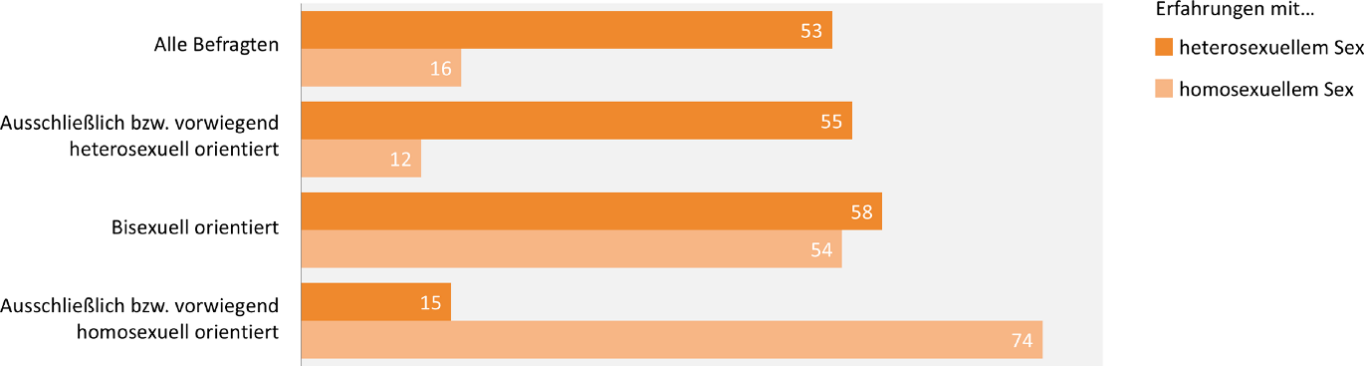
Sexuelle Kontakte in Abhängigkeit der sexuellen Orientierung in der 10. Befragungswelle der Jugendsexualitätsstudie 2025. (Fragen: Es gibt verschiedene Arten des Austausches von Zärtlichkeiten zwischen Mann und Frau. Bitte markieren Sie alles, was Sie hiervon selbst schon einmal gemacht oder erlebt haben. HIER: heterosexueller Sex//Es gibt ja auch verschiedene Arten des Austausches von Zärtlichkeiten zwischen Menschen gleichen Geschlechts. Bitte markieren Sie alles, was Sie hiervon selbst schon einmal gemacht oder erlebt haben. HIER: Kontakte über Küssen/Streicheln hinaus | Basis: 14- bis 25-Jährige (*N* = 5855, ungewichtet) | Darstellung: Angaben in Prozent)

Die sexuelle Praxis entspricht überwiegend der sexuellen Orientierung, der sich die Jugendlichen und jungen Erwachsenen zuordnen. Bei den Befragten, die eine (vorwiegend) homosexuelle oder heterosexuelle Orientierung angeben, zeigt sich, dass sie auch von sexuellen Erfahrungen außerhalb ihrer jeweils angegebenen Orientierung berichten (heterosexuelle Erfahrungen bei homosexuell orientierten Befragten und umgekehrt). Bei bisexuell orientierten Personen sind Erfahrungen mit beiden Geschlechtern annähernd gleich häufig (Abb. 1).

Den ersten Sex erlebt über die Hälfte der Befragten (58 % in 2025 gegenüber (ggü.) 56 % in 2019) mit dem Partner bzw. der Partnerin. Weitere 3 von 10 geben an, dass ihnen die Person gut bekannt gewesen sei (28 % in 2025 ggü. 28 % in 2019). Demgegenüber kannten nur wenige ihren Sexualpartner bzw. ihre Sexualpartnerin lediglich flüchtig (9 % in 2025 ggü. 16 % in 2019).

#### Beginn körperlicher Annäherungen und (hetero-)sexueller Aktivitäten

**Abb. 2:**
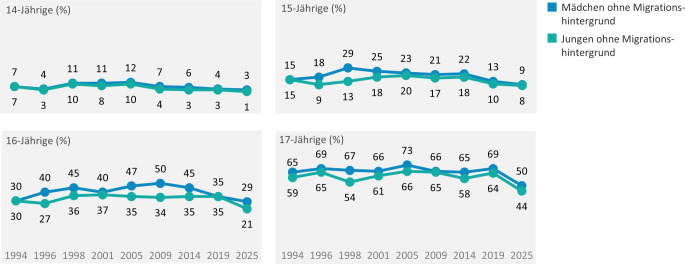
Erfahrung mit heterosexuellem Sex nach Alter: Trendvergleich der Befragungswellen der Jugendsexualitätsstudie 1994 bis 2025. (Frage: Bitte markieren Sie alles, was Sie hiervon selbst schon einmal gemacht oder erlebt haben. HIER: heterosexueller Sex | Basis: 14- bis 17-Jährige ohne Migrationshintergrund (*N* = 2487, ungewichtet) | Darstellung: Angaben in Prozent)

Die Daten belegen eine weitere Verschiebung des ersten heterosexuellen Sex von Jugendlichen auf einen späteren Zeitpunkt (Abb. 2 für die Darstellung des Langzeittrends der Jugendlichen ohne Migrationshintergrund). Eine Betrachtung aller Befragten zeigt, dass diese Verschiebung über alle Altersgruppen hinweg zu beobachten ist und auch junge Erwachsene einschließt. Im Vergleich zur Vorwelle aus dem Jahr 2019 ist insbesondere in den Altersjahren 17 bis 20 ein deutlicher Rückgang des Anteils derjenigen zu verzeichnen, die Erfahrungen mit heterosexuellem Sex gemacht haben (Rückgang um 16 bis 22 Prozentpunkte). Während in der vorangegangenen Erhebungswelle noch 61 % der 17-Jährigen angaben, bereits heterosexuellen Sex erlebt zu haben, liegt dieser Anteil in der aktuellen Erhebungswelle nur noch bei 40 %.

Mit zunehmendem Alter steigt die sexuelle Aktivität: Mehr als die Hälfte der 19-Jährigen berichtet von ersten heterosexuellen Erfahrungen (59 % im Jahr 2025 ggü. 75 % im Jahr 2019). In den Altersgruppen der 23- bis 25-Jährigen geben etwa 8 von 10 jungen Erwachsenen an, heterosexuellen Sex gehabt zu haben (81–82 % im Jahr 2025 ggü. 89–90 % im Jahr 2019). Bezogen auf Erfahrungen mit homosexuellem Sex sind keine direkten Zeitreihenvergleiche zu früheren Befragungswellen möglich (siehe Abschn. Diskussion).

Auch hinsichtlich anderer Formen heterosexueller Kontakte berichten Jugendliche und junge Erwachsene von weniger Erfahrungen im Vergleich zur vorangehenden Erhebung. So gaben 2019 noch 53 % der 14-Jährigen an, Erfahrungen im Küssen gemacht zu haben; in der aktuellen Erhebungswelle ist dieser Anteil deutlich auf 33 % gesunken. Unter den 15-Jährigen hat rund die Hälfte (51 %) den ersten Kuss erlebt – auch dies entspricht einem Rückgang um 19 Prozentpunkte gegenüber der vorherigen Welle. Mit zunehmendem Alter verringern sich die Abweichungen zur Vorwelle; dennoch liegen die aktuellen Werte in sämtlichen Altersjahren zwischen 2 und 20 Prozentpunkten unter den Vergleichswerten aus 2019. Auch Erfahrungen mit Intimpetting (von männlicher und weiblicher Seite) sind in den verschiedenen Altersgruppen rückläufig – am deutlichsten zeigt sich dieser Rückgang bei den 17- bis 20-Jährigen, mit einer Abnahme von 13 bis 22 Prozentpunkten gegenüber der vorherigen Erhebungswelle.

Als entscheidende Gründe dafür, keine körperlichen Annäherungen einzugehen, geben sexuell inaktive Jugendliche und junge Erwachsene vor allem an, dass ihnen bislang „der richtige Partner bzw. die richtige Partnerin fehlte“ (Jugendliche: 51 %, junge Erwachsene: 53 %) und dass sie „zu schüchtern“ seien (Jugendliche: 37 %, junge Erwachsene: 33 %). Für sexuell inaktive Jugendliche ist zudem ausschlaggebend, dass sie „noch zu jung“ (41 %) seien.

### Kontrazeptionsverhalten

#### Verhütung beim ersten heterosexuellen Sex

**Abb. 3:**
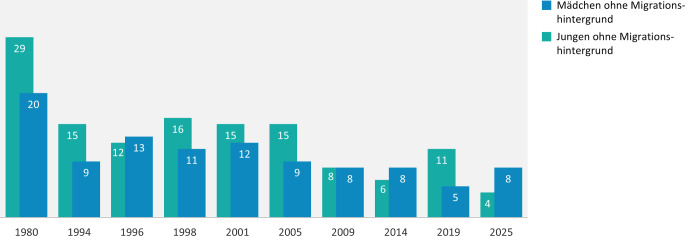
Keine Verhütung beim ersten heterosexuellen Sex: Trendvergleich der Befragungswellen der Jugendsexualitätsstudie 1980 bis 2025. (Frage: Was haben Sie und/oder Ihr Partner/Ihre Partnerin beim ersten heterosexuellen Sex unternommen, um eine Schwangerschaft zu verhüten? HIER: nichts unternommen | Basis: 14- bis 17-Jährige ohne Migrationshintergrund mit heterosexueller Sexerfahrung (*N* = 512, ungewichtet) | Darstellung: Angaben in Prozent)

Beim ersten heterosexuellen Sex geben 5 % der 14- bis 17-Jährigen an, nicht verhütet zu haben. Abb. 3 zeigt den Langzeittrend der 14- bis 17-Jährigen ohne Migrationshintergrund, die bei ihrem ersten heterosexuellen Sex keine Verhütungsmethode verwendet haben. Bei den Jungen ist der Anteil der Nichtverhütenden im Vergleich zu 2019 um 7 Prozentpunkte gesunken und stellt mit 4 % den niedrigsten Wert seit 1980 dar. Bei den Mädchen ohne Migrationshintergrund ist der Anteil der Nichtverhütenden gegenüber der Vorwelle geringfügig gestiegen (um 3 Prozentpunkte) und liegt damit auf dem Niveau der anderen Befragungswellen der letzten 2 Dekaden. Mit 8 % geben aktuell etwa doppelt so viele Mädchen ohne Migrationshintergrund an, beim „ersten Mal“ nicht verhütet zu haben, wie gleichaltrige Jungen.

Ob 14- bis 17-Jährige bei ihrem ersten Sex auf Verhütung verzichteten, hängt auch davon ab, wie alt sie zum Zeitpunkt des ersten Sex waren: Waren die Jugendlichen bei ihrem „ersten Mal“ 14 Jahre oder jünger, geben sie mehr als doppelt so häufig an, nicht verhütet zu haben, wie Jugendliche, die bei ihrem ersten Sex 15 oder 16 Jahre alt waren (14-Jährige: 14 % vs. 15-Jährige: 5 % vs. 16-Jährige: 2 %). Aufgrund der zu geringen Fallzahl ist eine Auswertung zur (Nicht‑)Verhütung beim ersten homosexuellen Sex der männlichen Befragten nicht möglich (siehe Abschn. Diskussion).

#### Wahl der Verhütungsmethode

**Abb. 4:**
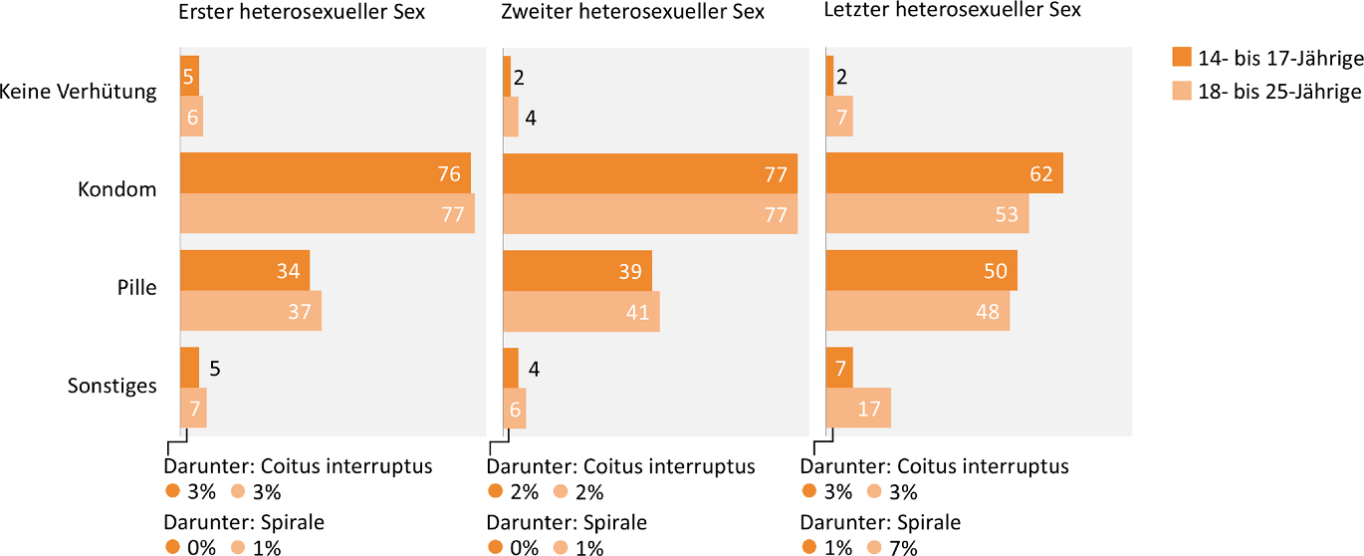
Verhütungsverhalten mit zunehmender Sexerfahrung in der 10. Befragungswelle der Jugendsexualitätsstudie 2025. (Frage: Was haben Sie und/oder Ihr Partner/Ihre Partnerin beim 1./2./letzten heterosexuellen Sex unternommen, um eine Schwangerschaft zu verhüten? | Basis: 14- bis 25-Jährige mit (mehrmaliger) heterosexueller Sexerfahrung (*N* = 2260 bzw. *N* = 2183 bzw. *N* = 2197, ungewichtet) | Mehrfachnennungen möglich | Darstellung: Angaben in Prozent)

Die Wahl der Verhütungsmethode hängt vom Alter der Befragten sowie ihrer sexuellen Erfahrung ab (Abb. 4).

Über Dreiviertel der Jugendlichen (76 %) geben an, beim ersten Sex mit Kondom verhütet zu haben (ggü. 77 % in 2019), während ein Drittel (34 %) mit der Pille verhütete (ggü. 30 % in 2019). Mit zunehmender sexueller Erfahrung steigt auch die Verwendung der Pille durch Jugendliche, während die Verhütung mit Kondomen etwas abnimmt. Dennoch werden Kondome häufiger genutzt als die Pille: Bei ihrem letzten Sex nutzte die Hälfte der Jugendlichen (50 %) die Pille zur Verhütung (ggü. 53 % in 2019), während 62 % Kondome nutzten (ggü. 63 % in 2019). Junge Erwachsene verwendeten bei ihrem letzten Sex Pille und Kondom etwa gleich häufig (2025: Pille: 48 %; Kondom: 53 %; ggü. 2019: Pille: 59 %; Kondom: 48 %).

Die Pille wird vor allem von Befragten in festen Beziehungen als Verhütungsmethode bevorzugt (54 %), während sie seltener von Befragten in anderen Beziehungsformen (37 %) oder ohne Partnerschaft (40 %) genutzt wird. Umgekehrt verwenden Befragte in anderen Beziehungsformen (64 %) oder ohne Partnerschaft (69 %) häufiger Kondome als Befragte in festen Beziehungen (44 %).

Insgesamt sind die Pille und das Kondom die am meisten verwendeten Verhütungsmethoden für junge Menschen. Lediglich 7 % der Jugendlichen geben an, bei ihrem letzten Sex alternative Verhütungsmethoden zu Pille und Kondom verwendet zu haben. Bei den jungen Erwachsenen sind es mit 17 % mehr als doppelt so viele, wobei am häufigsten die Spirale genannt wird (7 %).

#### Nutzung von und Einstellung zu hormoneller Verhütung

Die Pille ist die am häufigsten genutzte Verhütungsmethode von sexuell aktiven Mädchen und jungen Frauen in festen Partnerschaften: Mehr als die Hälfte (56 %) von ihnen verwenden sie zur Verhütung. Ein Vergleich der Pillennutzung seit 2014 zeigt einen insgesamt rückläufigen Trend, der sich sowohl bei Personen in fester Partnerschaft (Verhütung beim letzten Sex: 54 % in 2025 ggü. 65 % in 2019 und 78 % in 2014) als auch – in noch stärkerem Maße – bei Personen ohne Partnerschaft beobachten lässt (Verhütung beim letzten Sex: 40 % in 2025 ggü. 54 % in 2019 und 66 % in 2014).

**Abb. 5:**
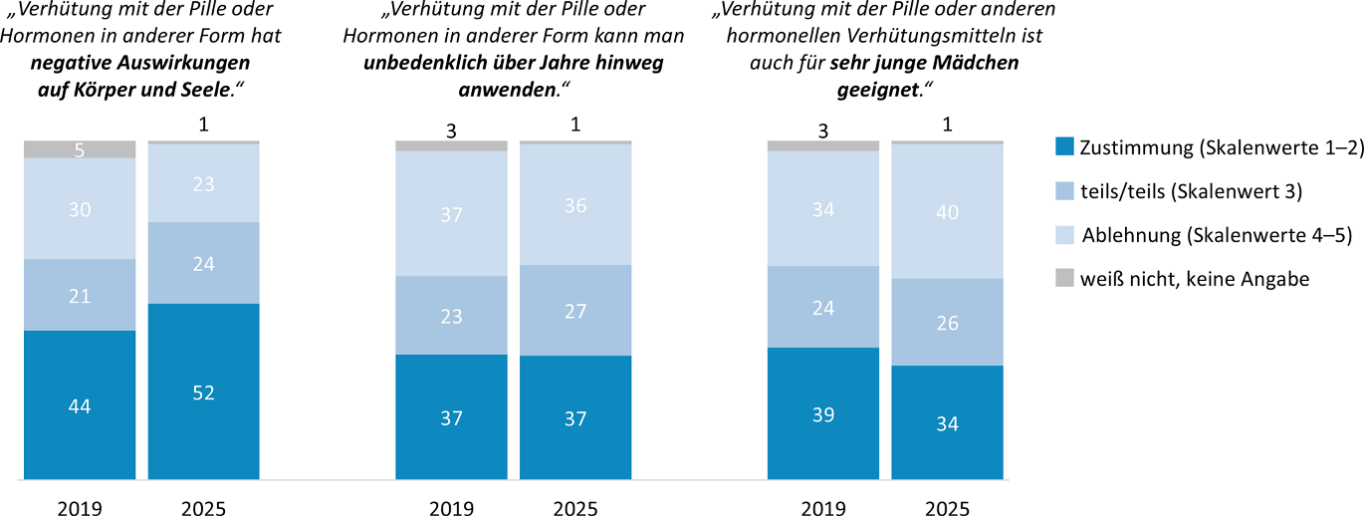
Einstellung zu hormoneller Verhütung: Vergleich der 9. und 10. Befragungswelle der Jugendsexualitätsstudie (2019 vs. 2025). (Frage: Zu Verhütungsmitteln gibt es ja unterschiedliche Ansichten. Auf einer Skala von 1 „stimme vollkommen zu“ bis 5 „stimme überhaupt nicht zu“: Wie sehr stimmen Sie persönlich den folgenden Ansichten zu? | Basis: 14- bis 25-jährige Mädchen/junge Frauen mit mehrmaliger heterosexueller Sexerfahrung (*N* = 1429, ungewichtet) | Darstellung: Angaben in Prozent „Top-Two-Werte“ (1–2, Zustimmung), mittlerer Skalenwert (3, teils/teils), „Bottom-Two-Werte“ (4–5, Ablehnung))

Die bereits im Jahr 2019 gegenüber 2014 festgestellte Zunahme an Skepsis gegenüber der Gesundheitsverträglichkeit der Pille setzt sich in der vorliegenden Befragung fort (Skala von 1 = sehr gut bis 6 = sehr schlecht: 2025: M = 3,3, Standardabweichung (SD) = 1,5; 2019: M = 3,08, SD = 1,51; 2014: M = 2,64; SD = 1,35). Wie in Abb. 5 erkennbar ist, äußern sich Mädchen und junge Frauen im Vergleich zur Vorwelle entsprechend häufiger hormonkritisch.

Eine eher kritische Haltung zeigt sich auch bei den 14- bis 25-jährigen Mädchen und jungen Frauen, die beim letzten Sex die Pille verwendet haben („aktive Pillennutzerinnen“): Mehr als jede Dritte (37 %) ist der Ansicht, dass die Pille oder andere hormonelle Mittel Körper und Seele schaden (Ablehnung: 37 %, unentschieden: 26 %). Zudem glauben 18 % nicht, dass man über Jahre hinweg unbedenklich mit Pille oder anderen hormonellen Kontrazeptiva verhüten kann (Zustimmung: 52 %, unentschieden: 28 %). 26 % der aktiven Pillennutzerinnen lehnen außerdem die Aussage ab, dass hormonelle Verhütungsmittel auch für sehr junge Menschen geeignet seien (Zustimmung: 48 %, unentschieden: 26 %).

#### Notfallkontrazeption

Insgesamt sind etwa 9 von 10 (92 %) der 14- bis 25-jährigen Mädchen und jungen Frauen über die Möglichkeit der Notfallverhütung mittels der „Pille danach“ informiert. Unter den sexuell aktiven Mädchen und jungen Frauen wissen nahezu alle von dieser Möglichkeit (98 % ggü. 96 % im Jahr 2019). Die „Pille danach“ ist auch den meisten Jungen und jungen Männern bekannt (80 %). Im Folgenden wird sich auf die Angaben der weiblichen Befragten zur Inanspruchnahme von Notfallverhütungsmitteln beschränkt.

Knapp ein Drittel der sexuell aktiven weiblichen Befragten hat mindestens einmal die „Pille danach“ zur Notfallverhütung eingesetzt (29 % in 2025 ggü. 27 % in 2019); 11 % griffen auch schon mehrmals darauf zurück (ggü. 9 % in 2019).

Die Anwendungshäufigkeit der „Pille danach“ nimmt mit zunehmendem Alter (und damit einhergehender sexueller Erfahrung) zu: Minderjährige heterosexuell aktive Mädchen berichten deutlich seltener von der Nutzung einer Notfallkontrazeption (einmal: 5 %; mehrmals: 4 %) als volljährige junge Frauen (einmal: 20 %; mehrmals: 11 %).

## Diskussion

Die 10. Welle der Jugendsexualitätsstudie belegt eine fortgesetzte zeitliche Verschiebung des heterosexuellen Erstkontakts nach hinten und – altersgruppenübergreifend – einen deutlichen Rückgang weiterer körperlicher Annäherungen wie Küssen und Intimpetting. Als Gründe geben junge Menschen das Fehlen der richtigen Partnerin/des richtigen Partners an und vor allem Jungen beschreiben sich häufig als zu schüchtern. Der erste hetero- und homosexuelle Sex erfolgt überwiegend in Partnerschaften.

Beim ersten heterosexuellen Sex ist Nichtverhütung selten, ein leichter Anstieg des Anteiles an nicht verhütenden jungen Frauen muss jedoch sorgfältig beobachtet und im Rahmen von Präventionsmaßnahmen adressiert werden. Des Weiteren zeigt sich ein erhöhtes Risiko für ausbleibende oder unsichere Verhütung beim ersten Sex vor allem bei sehr jungem Alter (≤ 14 Jahre). Das Wissen über die Notfallkontrazeption ist weitverbreitet und die Inanspruchnahme steigt erwartungsgemäß mit dem Alter an, wobei im Zeitverlauf keine Zunahme festzustellen ist. Da auch homo- und bisexuell orientierte Befragte zu einem nicht geringen Anteil von Erfahrungen mit heterosexuellem Sex berichten, sollten diese Zielgruppen bei der Prävention ungewollter Schwangerschaften adressiert werden.

Der beobachtete spätere Einstieg in heterosexuelle Aktivitäten und ein sicheres Verhütungsverhalten fügen sich in nationale und internationale Trendberichte ein. Für Deutschland wiesen vorangegangene Analysen dieser und anderer Wiederholungsbefragungen ein sicheres Verhütungsverhalten und eine insgesamt verantwortungsvolle Gestaltung früher Sexualkontakte nach [5, 9, 10]. Auch internationale Daten dokumentieren spätere oder vorsichtigere Einstiege in sexuelle Aktivitäten sowie eine hohe Verhütungsprävalenz [11–13]. Mögliche Gründe – jenseits eines möglichen pandemiebedingten Kohorteneffekts – sind veränderte Freizeit- und Mediennutzungsmuster, die Zunahme psychosozialer Belastungen, aber auch veränderte Normen und Erwartungen an Intimität [14–16]. Als ein Risikofaktor für Nichtverhütung beim ersten heterosexuellen Sex gilt ein junges Alter der weiblichen Jugendlichen [17, 18].

Die Wahl der Verhütungsmethode spiegelt differenzierte Abwägungen entlang von Schutzbedürfnissen, Verfügbarkeit und Partnerschaft wider. Kondome bieten sowohl Schutz vor Schwangerschaft als auch vor sexuell übertragbaren Infektionen (STI) und werden insbesondere in jungem Alter und abseits von festen Partnerschaften eingesetzt. Mit zunehmendem Alter und einer gestiegenen Wahrscheinlichkeit für Partnerschaften wird die Pille häufiger verwendet. Dieses Nutzungsmuster im Kontext Kontrazeption sowie der beschriebene Rückgang der Pillennutzung bestätigen damit erneut den Trend, der in den vergangenen Jahren gut dokumentiert wurde [5, 19, 20], auch wenn sich der Nutzungsrückgang in der vorliegenden 10. Befragungswelle weniger deutlich darstellt: Die aktuellen Daten deuten darauf hin, dass der Rückgang beim „ersten Mal“ nicht weiter fortschreitet (stabile Nutzung im Vergleich zu 2019), während beim letzten Sex der Anteil der Pillennutzung weiterhin niedriger ausfällt.

Parallel nimmt die hormonskeptische Haltung zu. Diese Ambivalenz zwischen Einstellung und Verhalten entspricht Befunden aus der Erwachsenenbevölkerung, die plausibel darstellen, dass trotz manifester hormonkritischer Einstellungen weiterhin hormonelle Kontrazeption verwendet wird (siehe auch Beitrag von Knittel et al. in diesem Themenheft). Zahlreiche Studien zur Online-Kommunikation belegen des Weiteren den Einfluss von Social Media auf die Verbreitung von hormonkritischen Einstellungen [21–23]. Vor diesem Hintergrund erscheint der Ausbau methodenneutraler, evidenzbasierter Beratung [24] ebenso bedeutsam wie die Stärkung der digitalen Gesundheitskompetenz junger Menschen.

Limitierend sei erwähnt, dass die Studie auf Selbstangaben basiert und sozial erwünschte Antworten trotz Selbstausfülleranteilen nicht ausgeschlossen werden können. Als Querschnittserhebung sind zudem Aussagen über Kausalzusammenhänge und in manchen Fällen Teilanalysen (z. B. Gründe für Nichtverhütung in jungem Alter) aufgrund kleiner Fallzahlen nicht möglich. Das Weiteren konnten Personen außerhalb der binären Geschlechterkategorien mangels amtlicher Referenzzahlen zum Zeitpunkt der Durchführung noch nicht separat gewichtet und damit nicht in die Analyse einbezogen werden. Zudem sind detailliertere Aussagen oder Zeitreihenvergleiche zu sexuellen Erfahrungen und dem Verhütungsverhalten von homo- und bisexuell orientierten Befragten nur begrenzt möglich. Einerseits ist die verfügbare Fallzahl für Alters- oder Geschlechtsvergleiche zu gering, andererseits wurden homosexuelle Kontakte im Fragebogen mit Blick auf die Gesamtlänge der Interviews vergleichsweise knapp erfasst. An dieser Stelle sei auf andere explorative Studien verwiesen, die die sexuellen und partnerschaftlichen Erfahrungen insbesondere von LSBTIQ*-Personen in den Blick nehmen (z. B. [25, 26], siehe auch Beitrag von Stehr und Wazlawik in diesem Themenheft).

## Fazit

Zusammenfassend zeigt sich, dass Jugendliche und junge Erwachsene in Deutschland ihre ersten sexuellen Erfahrungen überwiegend verantwortungsvoll, informiert und in festen Beziehungen gestalten. Gleichzeitig setzt sich der Trend hin zu einem späteren Einstieg in sexuelle Aktivitäten fort. Die Verhütungsquote beim ersten Sex ist unverändert hoch; Kondom und Pille sind die meistgenutzten Verhütungsmethoden, wobei die Skepsis gegenüber hormonellen Methoden weiter zunimmt.

Angebote zur Förderung der sexuellen und reproduktiven Gesundheit müssen evidenzbasiert, methodenneutral, zielgruppenspezifisch, digital kompetenzorientiert sowie anschlussfähig an die jeweiligen Lebensphasen erfolgen. Schulische Angebote zur sexuellen Bildung sollten nach Möglichkeit evidenzbasiert entwickelt, weiter ausgebaut und langfristig in der Lehramtsaus- und -fortbildung berücksichtigt werden. Die Jugendsexualitätsstudie liefert seit vielen Jahrzehnten eine zuverlässige Datengrundlage, um Kommunikationsangebote passgenau weiterzuentwickeln und die sexuelle und reproduktive Gesundheit auch der nachfolgenden Generation junger Menschen nachhaltig zu fördern.

## Supplementary Information

ESM1: Zusatzmaterial 1

## Data Availability

Die im Rahmen dieser Studie erhobenen Daten werden in einem öffentlichen Repositorium gemäß dem FAIR-Prinzip veröffentlicht, um die Ergebnisse der Wissenschaft zugänglich zu machen.

## Footnotes

1 https://www.uni-bremen.de/divergestool-projekt/divergestool-toolbox (zugegriffen: 28.11.2025).

2 Während z. B. in der Vorwelle 2019 im Fragebogen noch von heterosexuellem „Geschlechtsverkehr“ und homosexuellem „Intimkontakt“ die Rede war, wurde das Wording im aktuellen Fragebogen vereinheitlicht zu „heterosexuellem bzw. homosexuellem Sex“.

3 Für Informationen zu Fragestellung, Antwortmöglichkeiten und Datenbasis der im Folgenden vorgestellten Ergebnisse siehe Tabelle Z1 im Onlinematerial.
