# Supplementary material for: Sexual and contraceptive behavior of adolescents and young adults in Germany: current results of the representative survey “Youth Sexuality”
Source: Bundesgesundheitsblatt Gesundheitsforschung Gesundheitsschutz. 2026 Feb 23;69(4):391–9. [Article in German] doi: 10.1007/s00103-026-04203-z (PMC13043589; doi:10.1007/s00103-026-04203-z)
Supplement: Supplementary file 1 — ESM1: Zusatzmaterial 1 [file 103_2026_4203_MOESM1_ESM.pdf]

Bundesinstitut für
Öffentliche Gesundheit

Tabelle Z1: Fragestellungen, Antwortmöglichkeiten und Datenbasis der 10. Befragungswelle der Jugendsexualitätsstudie 2025

**Fragestellungen**	**Antwortmöglichkeiten**	**Datenbasis**
Wählen Sie bitte die Antwort, die am besten beschreibt,<br>wie Sie momentan über sich selbst denken.<br><br>Ich bin …	1: ausschließlich heterosexuell<br>2: vorwiegend heterosexuell<br>3: bisexuell<br>4: vorwiegend homosexuell<br>5: ausschließlich homosexuell<br>6: asexuell<br>7: anderes, und zwar:<br><br>9: keine Angabe	14- bis 25-Jährige<br>(n = 5.855, ungewichtet)
Wie genau kannten Sie die Person, mit der Sie zum ersten Mal Sex hatten?	1: Die Person war mir vorher überhaupt nicht bekannt<br>2: Die Person war mir flüchtig bekannt<br>3: Die Person war mir gut bekannt<br>4: ich war mit der Person in einer Beziehung/zusammen<br>5: ich war mit der Person verlobt<br>6: ich war mit der Person verheiratet/in einer eingetragenen Lebenspartner-schaft<br><br>9: keine Angabe	14- bis 25-Jährige mit (hetero- und/oder homosexueller) Sex-Erfahrung<br>(n = 2.482, ungewichtet)
Es gibt verschiedene Arten des Austausches von Zärtlichkeiten zwischen Mann und Frau. Bitte markieren Sie alles, was Sie hiervon selbst schon einmal gemacht oder erlebt haben.	Mehrfachnennungen, Listenvorlage<br><br>11: Küssen<br>12: ein Junge/Mann streichelt die Brust eines Mädchens/einer Frau<br>13: Intimpetting von männlicher Seite: ein Junge/Mann berührt die Geschlechtsteile eines Mädchens/einer Frau<br>14: Intimpetting von weiblicher Seite: ein Mädchen/eine Frau berührt die Geschlechtsteile eines Jungen/Mannes<br>15: heterosexueller Sex	(HIER: heterosexueller Sex)<br>14- bis 17-Jährige ohne Migrationshintergrund<br>(n = 2.487, ungewichtet)<br>bzw.<br><br>alle Befragten<br>(n = 5.855, davon n = 3.514 Jugendliche und n = 2.341 junge Erwachsene, ungewichtet)

	98: nichts davon<br>99: keine Angabe	
Hier ist nun eine Liste mit Gründen, die viele genannt haben, weshalb sie keinen engeren Kontakt mit anderen Menschen gehabt haben. Markieren Sie bitte die Gründe, die für Sie hierbei entscheidend waren.	Mehrfachnennungen, Listenvorlage<br><br>11: mir fehlte bisher der richtige Partner/die richtige Partnerin<br>12: mein Interesse daran ist einfach zu gering<br>13: ich bin zu schüchtern<br>14: dafür bin ich noch zu jung<br>15: dadurch bindet man sich zu sehr<br>16: ich habe Angst, dass ich mich zu ungeschickt anstelle<br>17: ich finde es unmoralisch<br>18: der Partner/die Partnerin weigerte sich<br>19: weil meine Eltern es erfahren könnten<br>20: vor der Ehe finde ich das nicht richtig<br>21: ich muss erst mal auf eigenen Füßen stehen<br>22: ich habe Angst vor einer Schwangerschaft<br>23: ich fühle mich nur/mehr zu Personen meines Geschlechts hingezogen<br>24: ich habe schlechte Erfahrungen mit körperlicher Nähe gemacht<br>25: ich glaube, dass niemand körperlichen Kontakt zu mir haben möchte<br><br>99: keine Angabe	14- bis 25-Jährige ohne Erfahrungen im Austausch von Zärtlichkeiten in Abhängigkeit der eigenen sexuellen Orientierung (n = 1.344 Jugendliche und n = 233 junge Erwachsene, ungewichtet)
Was haben Sie und / oder Ihr Partner bzw. Ihre Partnerin beim ersten heterosexuellen Sex unternommen, um eine Schwangerschaft zu verhüten?	Mehrfachnennungen, Listenvorlage<br><br>11: keine Verhütungsmittel, nichts unternommen<br>12: Pille<br>13: Kondom<br>14: Hormonspirale	(HIER: keine Verhütungsmittel, nichts unternommen)<br>14-bis 17-Jährige, deren erster Sex heterosexuell war (n = 650, ungewichtet) bzw.

	15: Kupferspirale, Kupferkette, Kupferball<br>16: Zyklus-App, Kalendermethode („Tage-Zählen“)<br>17: Temperaturmethode<br>18: Natürliche Familienplanung (kurz „NFP“: Kombinierte Anwendung der Temperatur- und Kalendermethode sowie Zervixschleim-Beobachtung)<br>19: Vaginalring<br>20: Diaphragma<br>21: Verhütungsspritze, Dreimonatsspritze<br>22: Verhütungsstäbchen, Hormonimplantat<br>23: Verhütungspflaster<br>24: Chemische Verhütungsmittel (Spermizide)<br>25: „Aufpassen“ beim Sex, „rechtzeitiger Abbruch“ des Verkehrs („Coitus interruptus“)<br>31: Sterilisation des Mannes<br>32: Sterilisation der Frau<br><br>98: Sonstiges<br>99: keine Angabe	n = 117 Jugendliche, die ihr „erstes Mal“ im Alter von 14 Jahren oder jünger hatten und n = 469 Jugendliche, die ihr „erstes Mal“ mit 15 oder 16 Jahren hatten (ungewichtet)
Was waren die Gründe dafür, dass Sie bei Ihrem ersten Sex nicht verhütet haben?	Mehrfachnennungen, Listenvorlage<br><br>11: es kam so spontan, dass wir an nichts mehr dachten<br>12: ich habe daran gedacht, mich aber nicht getraut, es auszusprechen<br>13: ich hatte Alkohol/Drogen zu mir genommen und deshalb keinen klaren Kopf mehr<br>14: ich dachte, es wird schon nichts passieren<br>15: ich habe daran gedacht, es waren aber keine Verhütungsmittel verfügbar<br>16: ich habe mich nicht getraut, Kondome zu kaufen<br>17: ich hatte Angst zum Arzt/zur Ärztin zu gehen	Jugendliche, die beim ersten heterosexuellen Sex nicht verhütet haben (n < 100, ungewichtet)

	18: wir hatten uns vorgenommen, „aufzupassen“<br>19: ich/wir wusste(n) noch nicht so genau über Verhütung Bescheid<br>20: ich glaubte, dass ich mich auf meinen Partner/meine Partnerin verlassen kann<br>21: ich habe/meine Partnerin hatte vergessen, die Pille zu nehmen<br>22: zu teuer, wir konnten uns Verhütung nicht leisten<br>23: ich wollte verhüten, aber mein Partner/meine Partnerin war dagegen<br><br>97: anderer Grund (Welcher anderer Grund? Bitte kurz beschreiben)<br>98: ich weiß nicht, warum wir nicht verhütet haben<br>99: keine Angabe	
Welche Verhütungsmaßnahmen haben Sie und/oder Ihr Partner bzw. Ihre Partnerin beim letzten Sex angewendet?	Mehrfachnennungen, Listenvorlage<br><br>11: keine Verhütungsmittel, nichts unternommen<br><br>12: Pille<br>13: Kondom<br>14: Hormonspirale<br>15: Kupferspirale, Kupferkette, Kupferball<br>16: Zyklus-App, Kalendermethode („Tage-Zählen“)<br>17: Temperaturmethode<br>18: Natürliche Familienplanung (kurz „NFP“: Kombinierte Anwendung der Temperatur- und Kalendermethode sowie Zervixschleim-Beobachtung)<br>19: Vaginalring<br>20: Diaphragma<br>21: Verhütungsspritze, Dreimonatsspritze	14- bis 25-Jährige mit mehrmaliger heterosexueller Sex-Erfahrung (n = 606 Jugendliche und n = 1.591 junge Erwachsene) bzw.<br><br>n = 1.373 in einer festen Beziehung, n = 157 in anderen Partnerschafts-formen und n = 667 in keiner Partnerschaft (ungewichtet) bzw.<br><br>(HIER: Pille): 14- bis 25-jährige Mädchen und junge Frauen mit mehrmaliger heterosexueller Sex-Erfahrung in festen Beziehungen (n = 936, ungewichtet)

	22: Verhütungsstäbchen, Hormonimplantat<br>23: Verhütungspflaster<br>24: Chemische Verhütungsmittel (Spermizide)<br>25: „Aufpassen“ beim Sex, „rechtzeitiger Abbruch“ des Verkehrs („Coitus interruptus“)<br>31: Sterilisation des Mannes<br>32: Sterilisation der Frau<br><br>98: Sonstiges<br>99: keine Angabe	
Im Folgenden wollen wir von Ihnen wissen, wie bestimmte Eigenschaften von Verhütungsmitteln von Ihnen eingeschätzt werden: Zum Beispiel, ob Verhütungsmittel leicht im Geschäft zu bekommen sind, ob sie sicher sind, oder wie sie sich beim Sex auswirken. [Nähere Erläuterung]<br><br>Bewerten Sie bitte jetzt die Pille.<br><br>(1) Wie gut zu bekommen?<br>(2) Wie sicher?<br>(3) Wie preisgünstig?<br>(4) Wie zu handhaben?<br>(5) Wie gesundheitsverträglich?<br>(6) Wie wirkt es sich beim Sex aus?	1: (1) sehr gut<br>2: (2)<br>3: (3)<br>4: (4)<br>5: (5)<br>6: (6) sehr schlecht<br><br>8: weiß nicht<br>9: keine Angabe	(HIER: Wie gesundheitsverträglich?)<br>14- bis 25-jährige Mädchen/Frauen mit mehrmaliger heterosexueller Sex-Erfahrung<br>(n = 1.429 weiblich, ungewichtet) bzw.<br><br>14- bis 25-jährige Mädchen/ Frauen mit mehrmaliger heterosexueller Sex-Erfahrung, die beim letzten Sex die Pille verwendet haben<br>(n = 782, ungewichtet)
Nach einer Verhütungspanne oder nach ungeschütztem Sex gibt es die Möglichkeit der Notfall-Verhütung, die sogenannte 'Pille danach'. [Nähere Erläuterung]<br>Wussten Sie, dass es so eine Pille gibt?	1: ja<br>2: nein<br><br>9: weiß nicht, keine Angabe	14- bis 25-jährige Mädchen/ Frauen<br>(n = 3.556, davon n = 1.484 mit heterosexueller Sex-Erfahrung, ungewichtet)<br><br>14- bis 25-jährige Jungen/ Männer<br>(n = 2.256, ungewichtet)
[Nähere Erläuterung]<br>Haben Sie die 'Pille danach' selbst schon angewendet?	1: ja, einmal<br>2: ja, mehrmals<br><br>3: nein<br><br>9: keine Angabe	14- bis 25-jährige Mädchen/ Frauen mit mehrmaliger heterosexueller Sex-Erfahrung

		(n = 1.429, davon n = 363 Mädchen und n = 1.066 junge Frauen, ungewichtet)
Es gibt ja auch verschiedene Arten des Austausches von Zärtlichkeiten zwischen Menschen gleichen Geschlechts. Bitte markieren Sie alles, was Sie hiervon selbst schon einmal gemacht oder erlebt haben.	Mehrfachnennungen, Listenvorlage<br><br>11: Küssen/Streicheln<br>12: Kontakte über Küssen/ Streicheln hinaus<br><br>13: weder, noch<br><br>99: keine Angabe	(HIER: Kontakte über Küssen/Streicheln hinaus)<br>Alle Befragten<br>(n = 128 mind. vorwiegend homosexuell; n = 5.313 mind. vorwiegend hetero-sexuell, davon n = 4.818 ausschließlich heterosexuell; n = 290 bisexuell, ungewichtet)
